# GNAQ/GNA11-Related Benign and Malignant Entities—A Common Histoembriologic Origin or a Tissue-Dependent Coincidence

**DOI:** 10.3390/cancers16213672

**Published:** 2024-10-30

**Authors:** Justyna Pilch, Jakub Mizera, Maciej Tota, Piotr Donizy

**Affiliations:** 1 Department of Clinical and Experimental Pathology, Wroclaw Medical University, 50-556 Wroclaw, Poland; 2 Department of Pathology and Clinical Cytology, Jan Mikulicz-Radecki University Hospital, 50-556 Wroclaw, Poland

**Keywords:** uveal melanoma, GNAQ, GNA11, SOX10

## Abstract

Uveal melanoma (UM), recognized as the most prevalent primary intraocular malignancy in adults, is primarily driven by mutations in the *GNAQ* and *GNA11* genes. These genetic alterations are also implicated in other conditions, which exhibit distinct morphological characteristics. In this article, we investigate the role of *GNAQ* and *GNA11* mutations across varied disorders (e.g., UM, skin blue nevi, and hemangiomas), emphasizing the shared pathogenic mechanisms that connect them despite their differing clinical manifestations. By investigating the molecular pathways affected by these mutations, we provide insights into the potential for targeted therapies that could address not only UM but also other disorders associated with *GNAQ*/*GNA11* mutations. Moreover, we discuss the role of SOX10-positive perivascular cells that may be implicated in the complex pathophysiology of GNAQ/GNA11-related entities. Understanding the common molecular foundation of these conditions opens new ways for research and treatment opportunities, potentially leading to more effective, personalized therapeutic strategies.

## Simple Summary

Uveal melanoma (UM) is an aggressive cancer emerging from mutations in the *GNAQ* and *GNA11* genes. These mutations are also linked to other conditions with distinct morphological features (skin capillary malformations, hemangiomas, Sturge–Weber syndrome, choroidal nevi, blue nevi, hepatic small vessel neoplasm, Blitz nevi, iris and ciliary body melanocytoma, primary central nervous system melanocytic neoplasms, and aldosterone-producing adenomas). By examining the molecular pathways influenced by these mutations, we provide insights into potential targeted therapies for UM and related disorders. Additionally, we address the involvement of SOX10-positive perivascular cells in the complex pathophysiology of GNAQ/GNA11-related conditions.

## 1. Introduction

*GNAQ*/*GNA11* (*G protein subunits alpha q/11*) genes encode the alpha subunits of the heterotrimeric guanine nucleotide-binding proteins (G proteins), Gq and G11 [1]. G proteins are signal transducers that transmit signals from various neurotransmitters, hormones, chemokines, and other paracrine and autocrine factors. Interactions with this diversity of particles begin with the participation of the broad family of receptors with seven membrane-spanning regions, which then activate G proteins. Subsequently, signals can be routed to several distinct intracellular molecular pathways. These pathways cooperate to create a network responsible for the regulation of metabolic enzymes, transporters, ion channels, and other cellular machinery components. Moreover, they influence a wide range of cellular processes, including secretion, contractility, motility, and transcription. In turn, G proteins maintain organism homeostasis, and their dysfunction may lead to cellular proliferation and even oncogenesis [2,3]. Hence, due to the variety of functions controlled by G proteins, mutations in GNAQ and GNA11 genes may be found in a diversity of conditions, from benign lesions such as common blue nevi, which remain relatively neutral for individuals’ homeostasis, to aggressive malignant tumors with a poor prognosis like uveal melanoma [4,5].

Uveal melanoma (UM) is an aggressive malignancy originating from melanocytes in the eye, mainly affecting the choroid [4]. Even though it is the most common primary intraocular malignant neoplasm in adults, with approximately 5 cases per million per year, it can be classified as a rare cancer. Metastatic UM is an orphan disease that results in fewer therapeutic options and a lack of treatment guidelines. Due to molecular differences with cutaneous melanoma (CM), patients suffering from UM are often excluded from clinical trials regarding CM [6]. Early neoplasm detection is crucial for higher survival rates compared to patients with metastatic processes [7].

Some molecular findings regarding UM were made previously, and the role of genes *GNAQ* and *GNA11* in UM pathogenesis was highlighted as one of the most common molecular factors regarding this neoplasm [2,8]. Depending on the affected cell or the time of life when the mutation occurs, the same genetic changes can result in different phenotypic effects. Identical mutations can also be found in conditions such as skin blue nevi or hemangiomas [5]. Other mutations highly prevalent in UM are *BAP1* (*BRCA1-associated protein 1*), *EIF1AX* (*eukaryotic translation initiation factor 1A X-linked*), *SF3B1* (*splicing factor 3b subunit 1*), *CYSLTR2* (*cysteinyl-leukotriene receptor 2*), and *PLCB4* (*phospholipase C beta 4*) [9,10,11].

In the article, we attempt to comprise knowledge available from clinical trials and meta-analyses and assess whether there is any correlation between the occurrence of benign skin blue nevi or hemangiomas with uveal melanoma and other medical conditions caused by GNAQ and GNA11 mutations.

## 2. Biological Functions of *GNAQ* and *GNA11* Genes and Derived Proteins

### 2.1. GNAQ

**Figure 1:**
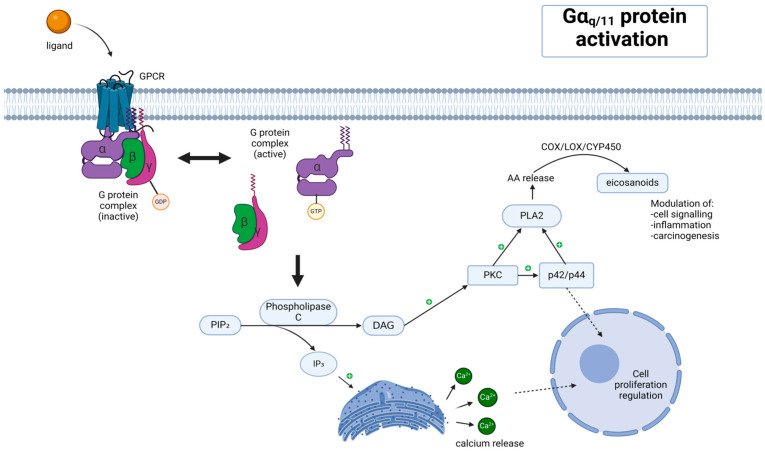
The signaling pathway associated with the activation of the Gαq/11 protein. A ligand binds to a GPCR (G-protein-coupled receptor) embedded in the cell membrane, triggering the activation of the G protein complex. The activated Gαq/11 subunit activates phospholipase C (PLC), which then catalyzes the hydrolysis of PIP_2_ (phosphatidylinositol 4,5-bisphosphate) into two secondary messengers: diacylglycerol (DAG) and inositol trisphosphate (IP_3_). IP_3_ binds to its receptors on the endoplasmic reticulum, leading to the release of Ca^2+^ ions into the cytoplasm. The increase in intracellular calcium concentration acts as a signal for various cellular processes. DAG remains in the membrane and activates protein kinase C (PKC),which, in turn, can phosphorylate various target proteins involved in cell signaling. PKC also activates phospholipase A2 (PLA2), catalyzing the release of arachidonic acid (AA) from membrane phospholipids, which is then metabolized by enzymes such as COX (cyclooxygenase), LOX (lipoxygenase), and CYP450 (cytochrome P450) to produce eicosanoids. Eicosanoids are involved in modulating processes like cell signaling, inflammation, and carcinogenesis. The pathway ultimately influences the regulation of cell proliferation. Specifically, the signaling cascade involving PKC and p42/p44 (ERK1/2) MAP kinases contributes to the control of cell growth and division. The symbol of a white plus inside a green circle in the figure should be understood as an activation.

*GNAQ* (*G protein subunit alpha q*) is a protein-coding gene located on chromosome 9 (9q21.2). It consists of 315,715 nt (nucleotides), including UTRs, with a total exon count of 7 [12]. Its aligned and CDS length amounts to 6882 nt and 1080 nt, respectively [13]. A study performed by Dong et al. proved that the GNAQ gene encodes 359 amino acids long protein, Gαq [14]. It is a guanine nucleotide-binding protein that constitutes a part of a trimeric G protein complex that mediates the stimulation of phospholipase C beta [5]. Guanine nucleotide-binding proteins form a family of heterotrimeric proteins coupling cell surface with 7-transmembrane domain receptors to signaling pathways inside a cell. The receptor activation process enables the catalysis of the exchange of GTP for GDP attached to the inactive G protein alpha subunit, causing a conformational change and dissociation of the complex. The activation process is terminated by a GTPase specific to the G-alpha subunit. Gαq, together with beta-gamma subunits, are proteins able to regulate various cellular effectors. They can manifest through various receptors, including the endothelin 1 receptor, vasopressin type 1A, and 1B receptors, angiotensin 2 receptor type I, α-1 adrenergic receptors, and others [14,15] [Figure 1].

Due to its essential cellular functions, the *GNAQ* gene is considered a proto-oncogene, especially referring to melanocytes and uveal melanomas. Nonetheless, *GNAQ* mutations play a significant role in other pathologies, such as Sturge–Weber syndrome, haemangiomas, or skin blue nevi [5]. They are concerned with codon Q209 and R183 hot spots. Interestingly, these mutations apply in the vast majority of mentioned pathologies (more than 80% of all cases) [1]. The Arg183Gln mutation, considering the *GNAQ* gene, affects the site of water autohydrolysis inside the GTP–GDP binding site of Gαq. Consequently, it is anticipated that the mutation reduces the effectiveness of autohydrolysis, which allows the guanine nucleotide protein to return to the deactivated (GDP-bound) state and complex with its GPCR (G protein-coupled receptor). Therefore, this mutation is hypothesized to result in constitutive overactivation of downstream pathways. As for the R183Q mutation, it was proved that mutant cells were characterized by a significant increase in phosphorylated extracellular signal-regulated kinase (ERK) compared to normal cells [16]. The issue is concerning since ERK plays an important role in regulating immune cell function as well as impacts cell growth, proliferation, cell differentiation, and apoptosis [17,18].

Furthermore, the same mutations mentioned above can result in vascular malformations, such as hemangiomas. However, it remains unclear why, in some cases, these mutations lead to carcinogenesis, while in others, they result in vascular malformations or benign hyperplasia. This variation is believed to depend on factors, such as the developmental stage at which the mutation occurs (germline vs. somatic), the specific cell type exposed to the mutation, or the presence of concurrent mutations. For instance, in aldosterone-producing adenomas, mutations in GNAQ and GNA11 remain clinically silent unless there is an accompanying mutation in the CTNNB1 gene [5]. Considering UM and blue nevi, germline mutations causing these conditions have not been discovered. Yet, there is supposed to be a hereditary factor that provokes the occurrence of familial melanoma, and it seems crucial to investigate the genomics of these individuals [19].

### 2.2. GNA11

*GNA11* (G protein subunit alpha 11) gene encodes the alpha subunit of G11 protein (Gα11)—an essential component of guanine nucleotide-binding protein complex (G proteins). The gene is located on chromosome 19p13.3 [20], and weighs 42,123 Da [21,22].

It was described by Strathmann and Simon while investigating the diversity of murine G protein [23]. Using methods based on PCR, they found evidence of a wide variety among G protein α subunits and divided them into three distinct groups: Gs, Gi, and Gq. Subunit Gα11 was classified into the Gq group along with the Gα14 [24]. In the same year, the human *GNA11* gene was cloned by Jiang et al., who found its product to be 359 amino acids long [25].

Heterotrimeric G11 protein, together with other G proteins, activates a complex network of crucial signaling pathways [1]. G11 protein, by cooperating with calcium-sensing receptors (CaSR), influences the processes responsible for regulating blood calcium levels [26]. Moreover, G11 signaling is involved in cell growth, proliferation, and apoptosis in various human tissues [27]. Hence, mutations in the GNA11 gene may result in diverse clinical disorders.

The *GNA11* gene may be affected by either germline or somatic mutations, both of them, are activating mutations, meaning that they lead to the production of an overactive Gα11 subunit of the G11 protein. In consequence, uncontrolled melanocyte proliferation in the uvea or the skin might be observed, as well as excessive inhibitory signaling, which blocks the increase in calcium levels despite them being below the normal range [28,29].

Germline mutations are generally inherited and occur in every cell of the body. In the case of *GNA11*, they result in various conditions associated with abnormal calcium concentration, such as autosomal dominant hypocalcemia type 2, also called autosomal dominant hypoparathyroidism due to inhibited production of parathyroid hormone (p.Arg60Leu, p.Arg60Cys, p.Arg181Gln, p.Ser211Trp, or p.Phe341Leu), or familial hypocalciuric hypercalcemia type 2 (p.Ile200del, p.Leu135Gln, or p.Thr54Met) [26,30,31].

Somatic mutations have been found in uveal melanomas [1]. Less commonly, they are associated with cutaneous or vascular lesions, such as blue nevus, [32], congenital extremity capillary malformation [33], Sturge–Weber syndrome [34], or cherry and congenital hemangioma [35,36].

**Figure 2:**
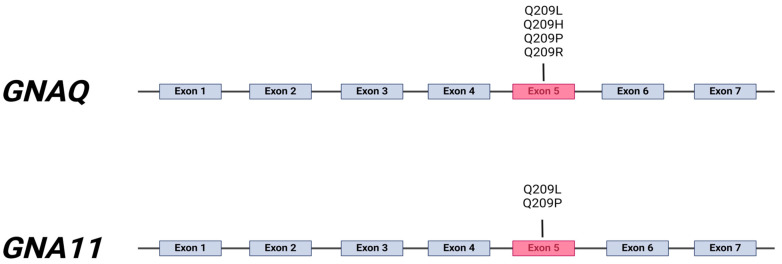
The most prevalent mutations within GNA genes—exon 5 in both *GNAQ* and *GNA11* appear to be a critical region, where specific mutations occur, particularly at the amino acid position Q209.

Due to their significant role in UM pathogenesis, *GNA11* somatic mutations are being further investigated in various research centers. A study conducted by Raamsdonk et al. provided detailed data on the prevalence of the two main somatic mutations found in both UM and blue nevi: mutation affecting codon Q209 (substitution of glutamine 209 for leucine or proline—p.Q209L and p.Q209P, respectively) were identified in 57% of uveal melanoma metastases, 32% of primary uveal melanomas, and 7% of blue nevi by gene sequencing of exon 5. Mutations in the same residue (Q209) of the paralogue gene *GNAQ* were found in 22% of uveal melanoma metastases, 45% of primary uveal melanomas, and 55% of blue nevi. Furthermore, sequencing of exon 4 of both *GNA11* and *GNAQ* genes, affecting codon R183 (arginine 183), showed a lower prevalence of mutations: 4.9% of primary uveal melanomas and 2.1% of blue nevi. The research evinced that these mutations were mutually exclusive, except for one tumor that carried them both—at Q209 and R138 in *GNA11*. Overall, 83% of all investigated uveal melanomas had oncogenic mutations in either *GNA11* or *GNAQ* [37] [Figure 2].

Notably, individuals with blue nevi or UMs seem to have normal blood calcium levels, and patients with an inherited *GNA11*-associated calcium concentration disorder do not appear to be at increased risk of developing either of these tumors [30]. This might result from the difference in underlying molecular pathogenesis, as different codons are affected by mutation within the same gene.

## 3. Disorders Associated with *GNAQ* and *GNA11* Mutations

### 3.1. Uveal Tract Tumors

#### 3.1.1. Choroidal Nevi

A choroidal nevus is a common and benign tumor arising from melanocytes in the choroid, often discovered accidentally during an ophthalmic examination. It can be found in 5–6.5% of the population and occurs more often in people with higher BMI and whites. Moreover, the lesion is significantly less common in people with blond hair. An association has not been found between nevus occurrence and visual impairments, glaucoma, or cataracts [38,39]. Even though choroidal nevi are considered stable lesions, there is a slight probability (varying in the range of 0.78–7%) of their malignant transformation to uveal melanoma [40]. The transformation may be suspected by features such as the thickness of the lesion greater than 2mm, symptoms of flashes or blurred vision, subretinal fluid, margin less than 3mm from the optic disc, orange lipofuscin pigment, halo or drusen absence, and ultrasonographic hollowness. If three or more of these features are present, there is more than a 50% probability of transformation to melanoma within five years [39]. Hence, all patients with choroidal nevi require long-term ophthalmological follow-up [41].

It is well known that rarely melanoma can develop from pre-existing nevus, which refers to CM arising from cutaneous nevi and UM arising from choroidal nevi. Vader et al. conducted research on post-mortem human eye samples to examine the pathomorphological basis of the condition. Molecular analysis revealed that mutations in choroidal nevi occur in both *GNAQ* and *GNA11* genes. Hotspots include Q209P and Q209L for the *GNAQ* gene and Q209L for the *GNA11* gene (Table 1). The vast majority of samples presented one out of three of these hotspot mutations, and the most commonly mutated hotspot was Q209L in *GNA11* [42]. Interestingly, identical mutations can be found in UM, clarifying the linkage between these two conditions [43]. The potential of choroidal nevi for its malignant transformation may be explained by the oncogenic features of *YAP*, which are already associated with several other malignancies. Mutant *GNAQ* and *GNA11* may result in *YAP* activation and, as a consequence, can lead to cancer formation [29].

#### 3.1.2. Uveal Melanoma

Mutations in the *GNAQ* and *GNA11* genes play a crucial role in the development of UM. In contrast to cutaneous melanomas, patients with UM rarely exhibit mutations in the frequently altered BRAF, NRAS, or NF1 genes [44,45]. Instead, *GNAQ* and *GNA11* mutations dominate, being identified in approximately 80% of cases [1,46]. The most frequent mutations in *GNAQ* and *GNA11* occur at codon 209 in exon 5 (Q209P, Q209L), which is a critical site for the protein’s GTPase activity [45]. These mutations result in impaired GTP hydrolysis, locking the Gα subunit in its active, GTP-bound form. This persistent activation leads to the constitutive stimulation of downstream signaling pathways, independent of external regulatory factors, thereby facilitating unregulated cellular proliferation and contributing to tumorigenesis [1]. Moreover, mutations at codon 183 (R183Q) can occur, although they are less common and typically exhibit reduced oncogenic potential compared to Q209 mutations [47]. The constitutive activation of *GNAQ* or *GNA11* via Q209 or R183 mutations leads to the sustained activation of multiple oncogenic pathways, such as the MAPK/ERK pathway, the Hippo/Yap-TAZ pathway, and the PI3K/AKT pathway [48,49,50].

Although mutations in both *GNAQ* and *GNA11* are present in primary UM (Table 1), research indicates that *GNA11* mutations, especially Q209L, have a stronger correlation with metastasis, particularly to the liver, which is the primary site for UM metastases [51]. About 50% of patients with UM develop metastatic disease, and the presence of *GNA11* mutations is frequently associated with a poor prognosis. Once metastasis has occurred, the disease is usually fatal, with few effective treatment options available [51].

The identification of *GNAQ* and *GNA11* mutations in UM has led to advancements in targeted therapies; however, direct inhibitors of these proteins have yet been developed. Present therapeutic strategies concentrate on inhibiting the downstream pathways activated by these mutations, using MEK inhibitors, YAP–TEAD pathway inhibitors, and PKC inhibitors [52,53,54]. Aforementioned treatment strategies are described in detail in Section 4.

#### 3.1.3. Iris and Ciliary Body Melanocytoma

Iris and ciliary body melanocytomas (magnocellular nevi) are rare and unusual melanocytic tumors. Although these tumors are benign, due to their proliferative nature, they may cause ophthalmic symptoms, such as elevated intraocular pressure and glaucoma. However, lesions are most commonly asymptomatic, with clinical presentation of dark spots involving the ciliary body and iris. Melanocytomas require a differential diagnosis with melanoma because of their macroscopic resemblance. Moreover, melanocytoma can sometimes transform into melanoma. Most commonly, lesions can be distinguished by histopathologic criteria after surgical procedures, such as iridocyclectomy [55,56].

Iris and ciliary body melanocytomas were examined for *GNAQ* and *GNA11* missense mutations. In the research performed by Solomon et al., in which 16 melanocytomas were analyzed, 15 of them demonstrated mutated genes—10 with a mutation in hotspot Q209P in *GNAQ* and 7 with a mutation in hotspot Q209L in *GNA11* [57]. Other researchers, such as Francis et al. and Mudhar et al., also confirmed that *GNAQ* mutations (codon Q209L) are present in iridociliary and other intraocular melanocytomas (Table 1) [58,59]. 

#### 3.1.4. Circumscribed Choroidal Hemangioma

Circumscribed choroidal hemangioma (CCH) is a benign vascular lesion, typically manifesting as a solitary orange-red mass localized at the posterior pole of the fundus. It is composed of an irregular, dilated, and congestive network of choroidal vessels separated by an intervascular septum. Unlike in the case of “true hemangiomas”, CCH cells do not proliferate, and the tumor tends to present slow or no progression in size over time [60]. 

Since CCH usually presents no symptoms until adulthood, it is rarely detected before vision impairment occurs. Generally, it manifests between the second and fourth decades of life as visual problems caused by intraretinal or subretinal fluid within the macula region or exudative retinal detachment [61]. In most cases, diagnosis is established based solely on clinical findings and eventually on the results of fluorescein angiography (FLA). FLA is performed in order to distinguish CCH from other tumors, such as choroidal metastasis or uveal melanoma. Differential diagnosis must also include diffuse choroidal hemangioma associated with Sturge–Weber Syndrome (SWS) and usually detected during childhood [62].

To determine the presence of oncogenic alterations of *GNAQ* and *GNA11* genes in CCH and their mutual profile, Le Guin et al. performed deep amplicon and Sanger sequencing of *GNAQ* and *GNA11* in DNA extracted from 33 samples diagnosed as CCH based on histological features. In 85% of the tissue samples (28/33), the identical *GNAQ* (p.Q209R) mutation was identified (Table 1). This narrow mutation spectrum of circumscribed choroidal hemangioma, restricted to *GNAQ* p.Q209R, may support researchers in establishing the diagnosis of CCH when clinical criteria are not sufficient or not enough biopsy samples are available to perform histological examination [63].

In the remaining five tissue samples, signals for oncogenic alleles at *GNAQ*/*GNA11* genes (p.Q209 or p.R183) were within the range of noise; hence, most probably, these samples do not have an oncogenic alteration at these sites. However, it is essential to consider that oncogenic activation of a G protein alpha subunit may occur due to a mutation at a different locus within the *GNAQ*/*GNA11* genes or within a gene encoding a different subunit, such as GNA14 [63,64].

### 3.2. Skin Capillary Malformations

Skin capillary malformations (SCMs) are congenital anomalies characterized by irregularities in capillary and venous blood vessels, presenting primarily in the skin. These malformations may also occur in the leptomeninges and choroid of the eye, particularly associated with Sturge–Weber Syndrome (SWS). In contrast, non-syndromic capillary malformations, which are more commonly observed, typically remain localized to the skin and do not extend to the brain or ocular structures [65]. SCMs may occur either as an isolated lesion or in association with different clinical findings, such as pigmentary birthmarks (characteristic of phacomatosis pigementovascularis) or leptomeningeal angiomatosis and glaucoma (characteristic of SWS). The use of targeted genetic sequencing has revealed that some of the SCMs subclassified by Happle into the “capillary naevi group”—nevus flammeus (port-wine stain), nevus anemicus, and cutis marmorata—are associated with postzygotic somatic mutations at residue 183 in *GNAQ* and *GNA11* genes [66,67].

The most frequent missense mutation in SCMs is *GNAQ* p.Arg183Gln, followed by other mutations affecting codon 183: p.Arg183Gly and p.Arg183Leu (Table 1). Since *GNAQ* and *GNA11* share more than 90% amino acid identity, a missense mutation in *GNA11* p.Arg183Cys and p.Arg183His was also found in patients suffering from a diffuse capillary malformation causing overgrowth and localized within an extremity [33].

To delineate the phenotype of patients with SCMs harboring an activating mutation in *GNAQ* or *GNA11*, Cuoto et al. analyzed 32 cases and concluded that nevus flammeus was associated with both *GNAQ* and *GNA11* mutations, with a slightly higher frequency of alterations regarding *GNAQ*. On the other hand, nevus anemicus and cutis marmorata were associated with *GNA11* mutations, while in nevus roseus—another subtype of capillary lesion—none of the *GNA*-associated mutations were found [33].

What is important is that nevus anemicus never manifested itself as a single lesion—it was always associated with another cutaneous capillary malformation (mainly cutis marmorata), giving rise to a lesion called nevus vascularis mixtus [33,68].

### 3.3. Sturge–Weber Syndrome

Sturge–Weber syndrome (SWS) is an uncommon and noninherited condition characterized by aberrant vasculature in the brain, skin, and eyes. It affects approximately 1 out of 20,000–50,000 people [69]. Patients with SWS frequently develop facial capillary malformations (also called port-wine birthmarks). Moreover, due to the abnormal blood vessels in the eye, they may suffer from ophthalmic disorders, such as glaucoma. Interestingly, glaucoma can be present at birth or develop later. SWS is also linked with altered cerebral perfusion, which leads to a high risk of seizures, venous stroke, and stroke-like events, and both motor and cognitive impairments in patients [70].

Recent studies suggest that the activating R183Q *GNAQ* somatic mutation is the most frequent mutation underlying SWS (Table 1). Furthermore, researchers point out the role of *GNA11* and *GNB2* somatic mutations in disease pathogenesis [70]. 

SWS caused by either *GNAQ* or *GNA11* mutation is clinically different. In the original study on the genetics of SWS performed by Dompmartin et al., 88% (23/26 examined) of the patients had a *GNAQ* mutation. They manifested dark homogenous SCMs, with the usual occurrence of nodules and continuous soft tissue hypertrophy of the face, causing a noticeable dysmorphism [34].

All three of the *GNA11*-SWS patients in the study exhibited patch and reticulated CMs that were pale at birth but gradually darkened with time, with the unusual appearance of nodules and restricted hypertrophy. Furthermore, patients with *GNA11*-SWS received a delayed diagnosis compared to those with *GNAQ*-SWS due to the more limited radiological features detected in brain MRI. As a result, two separate entities can be highlighted, *GNAQ*-SWS and *GNA11*-SWS, each of which has a unique clinical presentation and course [34,71].

Since the molecular basis of the SWS is complex and the condition may manifest in variable phenotypes, identifying the genetic basis in each individual seems crucial due to the different prognoses. As pharmacological therapies are developed to properly treat patients with vascular malformations, changes in molecular pathophysiology might affect responsiveness to therapies, increasing the importance of such genetically related phenotypic diagnoses [34].

### 3.4. GNA-Mutated Hemangiomas

#### 3.4.1. Congenital Hemangiomas

Congenital hemangiomas are rare vascular tumors arising during prenatal development. Postnatally, they may evolve following one of two clinical patterns: “rapidly involuting congenital hemangioma” (RICH) if the tumor involutes quickly or “non-involuting hemangioma” (NICH) if it partially regresses and stabilizes [72,73].

RICH can be diagnosed prenatally and manifests in newborns as raised, solitary cutaneous tumor with ectatic vessels, telangiectasis, and a pale halo. The lesion usually has a gray-violaceous appearance, demonstrates fast flow, and may be associated with transient low-grade thrombocytopenia or congestive heart failure. In the absence of serious complications related to hemorrhagic ulceration or heart failure, the tumor spontaneously regresses within 6–14 months. It leaves a skin mark characterized by persistent fast-flow, subcutaneous atrophy, and dilated vessels. Moreover, RICH was found and described to occur in the liver, where it regresses spontaneously, similar to cutaneous lesions [36]. NICH is a purple-pink plaque-like tumor, well-circumscribed by a pale rim from unchanged skin, displaying coarse telangiectasia and fast flow. Contrary to RICH, no complete regression is observed throughout childhood, and NICH remains unchanged or slightly regressed. In the literature, some cases of RICH were described as failing to involute and transform into NICH [36,74]. Both types of congenital hemangioma should be differentiated from common infantile hemangiomas, which—contrary to RICH and NICH—tend to enlarge rapidly after birth and immunostain for GLUT1, a cell surface marker [75].

To understand the molecular pathogenesis of congenital hemangiomas, Ayturk et al. examined eight fresh frozen tissue samples from patients diagnosed with congenital hemangioma and performed mRNA sequencing. This research resulted in the identification of missense mosaic mutations leading to the alteration of glutamine at amino acid 209 (Glu 209) in both *GNAQ* (c.626A>T, c.626A>C, c.627A>C) and *GNA11* (c.626A>T) genes in all tested specimens (Table 1). Since mutations were mutually exclusive, 6/8 samples were found to be associated with *GNAQ* somatic mutations and 2/8 with *GNA11* somatic mutation. Furthermore, the fact that the same missense mutation (*GNAQ* c.626A>T and *GNA11* c.626A>T) was found in both RICH and NICH implies the influence of other factors (genetic, epigenetic, and environmental) on these tumors’ different postnatal behavior [36].

#### 3.4.2. Cherry Hemangioma

Cherry hemangioma, also known as Campbell de Morgan spot or senile hemangioma, is the most frequent benign vascular tumor in adults. The latter variant of this term originates from lesions’ epidemiology—cherry hemangioma is extremely rare before puberty and particularly common in elderly people, as age appears to be one of the most crucial etiologic factors in its prevalence. Other potential etiologic factors reported in the literature are exposure to chemicals (e.g., sulfur mustard gas and bromide) and hormones (cherry hemangioma was described to occur in pregnant women and spontaneously regress after delivery). Senile hemangiomas typically manifest as well-circumscribed, small red-to-violaceous papules, primarily localize on the trunk and upper limbs. Histologically, they are composed of thin-walled capillary vessels, often surrounded by hyalinized stroma [35].

The molecular pathogenesis of cherry hemangioma is still poorly understood. Both non-neoplastic (which indicates that lesion may represent non-neoplastic proliferations, characterized by an overgrowth of mature vascular structures that closely resemble dermal venules) and neoplastic theories have been proposed as a potential etiology of the tumor. The absence of Ki-67 staining in tumor cells forms a base for non-neoplastic theory, while RAS mutations identified in 20% of senile hemangiomas suggest that some of them have neoplastic nature [76,77]. However, some of these findings should not be interpreted as definitive scientific conclusions due to the limitation of a small sample size. Nevertheless, we believe that the results are promising and warrant further investigation.

The results of the research conducted by Liau et al., comprising the molecular analysis of 68 cherry hemangioma samples and 17 cherry hemangioma-like hemangioma samples, allowed authors to establish the neoplastic nature of these vascular tumors. Both types of specimens had equal *GNA* gene mutation rates (82%). *GNAQ*, *GNA11*, and *GNA14* (particularly *GNA14* Q205L) mutations, which were mutually exclusive, were identified in approximately half of the cherry hemangiomas and cherry hemangioma-like hemangiomas. These outcomes provided solid evidence that cherry hemangiomas should be classified as benign vascular neoplasms caused most commonly by a mutation in the *GNA14* gene [35].

#### 3.4.3. Anastomosing Hemangioma

Anastomosing hemangioma (AH) is a benign vascular tumor that may localize in the genitourinary system, adrenal glands, liver, digestive tract, and mediastinum [78,79]. Besides visceral sites, AH has a remarkable predilection for paravertebral soft tissues [80]. The lesion is typically well-circumscribed and does not manifest dissecting or destructive growth. Histologically, AH displays either non-lobulated or loosely lobulated proliferation of anastomosing sinusoid capillaries, lined by bland endothelial cells. These cells are characterized by hyperchromatic nuclei protruding into the lumen and a hobnail-like appearance. Within the tumors, mild cytological atypia may be observed together with eosinophilic hyaline globules (i.e., thanatosomes), extramedullary hematopoiesis, and fibrin thrombi. Approximately 50% of anastomosing hemangiomas also present intravascular growth. Thus, due to its histology, AH might be confused with angiosarcoma. However, multilayering is absent, the mitotic rate is very low, and no aggressive behavior in this neoplasm has been reported [78,79,80].

The molecular pathogenesis was investigated by Bean et al., who identified activating mutations in *GNAQ* (p.Gln209His, p.Gln209Leu, p.Gln209Pro) and *GNA14* (p.Gln205Leu) genes (Table 1), responsible for the development of AH [81,82,83]. This research confirmed the theory claiming that anastomosing hemangioma is characterized by a neoplastic nature. A follow-up study conducted by Liau et al. aimed to discover the role of *GNA11* mutations (p.Gln209Leu, p.Gln209His) in AH development since this gene shares the highest degree of homology with *GNAQ*. Sanger sequencing and MassARRAY analysis identified mutually exclusive mutations in exon 5 of *GNAQ*, *GNA14*, and *GNA11* genes in 20 of the 22 investigated tumors. No significant differences in phenotype and molecular features were noticed between *GNAQ-*, *GNA11-*, or *GNA14*-mutated tumors. The results of these studies implicate that the essential mechanism underlying the pathogenesis of anastomosing hemangioma is related to *GNAQ* mutations, as well as its paralogues: *GNA11* and *GNA14*. 

#### 3.4.4. Hepatic Small Vessel Neoplasm

Hepatic small vessel neoplasm (HSVN) is considered a benign or low-grade infiltrative vascular neoplasm of the liver, composed of thin-walled small vessels. Due to its infiltrative nature, HSVN can mimic angiosarcoma, but unlike this type of cancer, HSVN presents no cytologic atypia or increased proliferation [84].

Joseph et al. performed a targeted panel sequencing of 479 cancer genes and exome sequencing to investigate the molecular pathogenesis of HSVN. The study was conducted on 18 benign or low-grade hepatic vascular neoplasms, including eight HSVN. It resulted in the identification of activating hotspot mutations in 75% (6/8) of HSNV—*GNA14* (p.Q205L, 4/8) or *GNAQ* (p.Q209H, 2/8) (Table 1). Mutations in these two genes were mutually exclusive. The remaining two samples (25%) demonstrated identical, previously unknown missense mutation in *GNAQ* (p.G48L) [85].

Although HSVN is a recently described neoplasm that has yet to be investigated, obtained data suggest that it shares similar molecular pathogenesis to other vascular malformations, such as port wine stains, tufted angioma, anastomosing, and congenital hemangioma, since mutations in these lesions are localized in the *GNAQ*, *GNA11*, and *GNA14* genes [33,36,85].

### 3.5. Phacomatosis Pigmentovascularis

Phacomatosis pigmentovascularis (PPV) is a common term for a family of rare disorders characterized by the coexistence of melanocytic lesions and vascular abnormalities. Clinical manifestations of PPV conditions might be observed as merely cutaneous or multisystemic [86]. Based on cutaneous findings, in 2005, Happle grouped PPV into three phenotypically different categories: phacomatosis cesioflammea (associated with blue spots and nevus flammeus), phacomatosis spilorosea (associated with nevus spilus with pale-pink telangiectatic nevus), and phocomatosis cesiomarmorata (associated with blue spots and cutis marmorata telangiectatica congenita) [87]. In 2012, this classification was updated to include the fourth variant of PPV—phacomatosis melanorosea (associated with flag-like hyper-melanotic nevus) [88,89]. According to other classification, there are five types of PPV: (1) naevus flammeus with naevus pigmentosus et verrucosus; (2) naevus flammeus with aberrant mongolian spots, with or without naevus anaemicus; (3) naevus flammeus with naevus spilus or giant speckled lentiginous naevus, with or without naevus anaemicus; (4) naevus flammeus with aberrant mongolian spots and naevus spilus or giant speckled lentiginous naevus, with or without naevus anaemicus; and (5) cutis marmorata telangiectatica congenita and aberrant mongolian spots [90,91].

It was recently discovered that two of the PPV subtypes—cesioflammea and cesiomarmorata—may share identical mosaic somatic activating mutations in *GNAQ* (p.Arg183Gln) and *GNA11* (p.Arg183Cys, p.Arg183Ser) genes. These molecular findings place the above-mentioned disorders on a clinical spectrum comprising other *GNA11-* and *GNAQ*-related conditions and suggest recommendations for their management [86]. The molecular pathogenesis of spilorosea and melanorosea has yet to be identified [92].

The case study and literature review by Kumar et al. aimed to determine the optimal surveillance approach for extracutaneous complications in individuals with phacomatosis cesioflammea and cesiomarmorata. Similar to other disorders associated with *GNAQ* or *GNA11* mutations, important extracutaneous complications in PPV can be predicted based on the location of cutaneous lesions and ocular melanosis. According to their findings, most commonly, patients suffered from abnormalities of the eye (ocular melanoma, glaucoma) and of the brain (development delay, imaging abnormalities). Moreover, the analysis of the patterns of co-occurrence between diverse clinical findings led to the identification of a potential positive correlation between the occurrence of ocular melanoma and ocular pigmentation, facial vascular involvement and seizures with development delay, limb asymmetry with either superficial or deep venous malformations [86]. The conclusion arising from this analysis is that individuals with ocular melanosis should be regularly followed up by an ophthalmologist [93], while those with facial vascular involvement should be evaluated by a neurologist or undergo brain imaging in case of neurological findings [94].

### 3.6. Blue Nevus

Blue nevi are common and benign melanocytic neoplasms that demonstrate a variety of clinical and morphological patterns, including common/dendritic, cellular, and atypical cellular subtypes. Like other nevi, they mostly develop in the skin but occasionally affect the lymph nodes, where they can be mistaken for metastatic melanoma [95]. Blue nevi can be characterized by the proliferation of dermal dendritic melanocytes. The spectrum of lesions associated with blue nevi ranges from common blue nevi, pigmented epithelioid melanocytoma to melanoma arising in blue naevus (MBN). MBN represents a range of histopathological features and genetic anomalies [96]. Melanomas that arise in the setting of blue nevi share a similar mutational and histopathological profile with uveal melanoma. The majority of UMs have the characteristic *GNA11* or *GNAQ* mutations, with additional *BAP1* mutations or deletions linked to a higher probability of metastases and worse prognosis overall [97].

Available data suggest that blue nevi are mostly positive for *GNAQ* (hotspot Q209L and Q209P) and *GNA11* (hotspot Q209L) mutations (Table 1). Estimates point out the fact that somatic mutations in codon 209 of *GNAQ* may be present in up to 80% of blue nevi. However, studies even reported that all examined lesions represented either *GNAQ* or *GNA11* mutations [32,98].

To sum up, *GNAQ* and *GNA11* gene mutations seem important for prognostic relevance, but other genes, such as *BAP1*, should also be considered as they seem to play an important role in the emergence of ‘benign’ metastases of blue nevi in lymph nodes or progression to aggressive malignant blue nevi [99,100].

### 3.7. Blitz Nevus

Blitz nevi are morphologically thought to show the mixed phenotype of Spitz and blue nevi, so genomically, they can also represent features of these lesions. While most blue nevi demonstrate *GNAQ* or *GNA11* mutations, Spitzoid neoplasms are known from either *HRAS* mutation or translocation involving *ROS*, *BRAF*, *MET*, *ALK1*, *RET*, and *NTRK1* [101]. To analyze possible genetic resemblance between blitz nevi and blue nevi, Isales et al. conducted research using NGS to examine lesions for *GNAQ* and *GNA11* mutations. The study revealed that 57% of blitz nevi acquired somatic mutations of these genes, also characteristic of blue nevi. Moreover, it was proved that all cases positive for *GNAQ* and *GNA11* mutations were at the same time negative for mutations characteristic of Spitzoid neoplasms. Hence, researchers suggest that genomically, blitz tumors should be categorized as a subset of blue nevi. However, this phenomenon requires further investigation and studies on a larger group of individuals [101].

### 3.8. Aldosterone-Producing Adenomas

An aldosterone-producing adenoma (APA) is a benign tumor affecting adrenal glands. The tumor causes primary hyperaldosteronism (PA), leading to higher salt retention in kidneys than in healthy people and, as a consequence, can cause hypertension or atrial fibrillation. The exact prevalence of adenomas is unknown, but they are estimated that they account for 60% of cases of primary hyperaldosteronism and 5–15% of all cases of hypertension [102]. The identification of PA and APA is crucial for decreasing cardiovascular morbidity and mortality, but current diagnostic techniques lack sensitivity and specificity, highlighting the need for new approaches [103].

Mostly, APA has somatic mutations in genes responsible for the function of ion channels or transporters, which puts a spotlight on this issue as a possible sensitive or specific diagnostic feature. Current scientific findings identify the role of mutations in *GNA11* (p.Gln209Leu, p.Gln209His, p.Gln209Pro), *GNAQ*, and *CTNNB1* (Table 1). Yet, the frequency of these mutations in the aldosterone-producing cell clusters of healthy adrenal glands suggests an obligatory role of codriver mutations. Hence, without a mutation of *CTNNB1*, *GNAQ* and *GNA11* mutations remain clinically silent [104,105].

In some congenital lesions, the mutation of *GNAQ* and *GNA11* is mosaic and can be found in several disparate sites. *GNA11/Q* mutations may develop early and established circumstances for a second hit in *CTNNB1* to result in APA development. This theory is supported by examples of *GNA11*/*Q* mosaicism that have been observed, and by the isolated, distinct regions of *GNA11* mutation in the nearby hyperplastic zona glomerulosa [104,106].

### 3.9. Primary CNS Melanocytic Neoplasms

Primary melanocytic neoplasms of the central nervous system (CNS) are uncommon lesions originating from melanocytes of the leptomeninges. Based on the WHO classification, they include:

1. circumscribed meningeal melanocytic neoplasms: melanocytoma (benign and well-differentiated lesion) and melanoma (malignant);
2. diffuse meningeal melanocytic neoplasms: melanocytosis (Diffuse or multifocal benign proliferation of cytologically bland melanocytic cells originating from leptomeningeal melanocytes) and melanomatosis (diffuse or multifocal malignant proliferation of melanoma cells arising from leptomeningeal melanocytes, frequently exhibiting invasion into the central nervous system) [107,108].

Typically, tumors represent a morphology of darkly pigmented, solid masses, often involving necrotic or hemorrhagic areas, composed of sheets of pleomorphic cells with prominent nucleoli, numerous mitotic figures, and parenchymal invasion. Symptoms differ by the location of the melanocytic neoplasm. Back pain, muscle weakness, numbness, plegia, or urinary incontinence may be manifestations of spinal tumors, while seizures, subarachnoid hemorrhage, or focal neurological symptoms may be signs of an intracranial tumor [109].

Clinically, primary leptomeningeal melanocytic neoplasms (LMNs) must be distinguished from metastatic lesions, since the prognosis for individuals with metastatic disease is less favorable. Therefore, diagnostic methods for a primary CNS melanocytic tumor, such as molecular diagnostics, might be useful in clinical settings [110].

Primary CNS melanocytic tumors are frequently affected by mutations in the heterotrimeric G protein alpha subunits, *GNAQ* or *GNA11* [111]. Although the predictive value of mutations in G-protein genes *GNA11* and *GNAQ* in patients with primary CNA melanocytic neoplasms remains unknown, a lot of studies are held to find out if this feature might be useful for tumor diagnostics [112]. Researchers found out that LMNs share some genetic abnormalities with UMs, including *GNAQ* (p.Gln209Leu) and *GNA11* (p.Gln209Leu, p.Gln209Pro) mutations, but these mutations are less common in LMNs than in UMs. Mutations in *GNAQ* R183 and *GNA11* R183 were not found, implying that these mutations are uncommon in LMNs, similar to their rarity in UMs [113].

To sum up, all these findings, together with diagnostics of *BRAF*, *HRAS*, and *NRAS* hotspot mutations, may be useful in the differential diagnosis between primary CNS melanocytic neoplasms and metastases from a CM to the CNS. This seems particularly important due to the fact of very different prognosis of these conditions [113].

**Table 1:** A summary of medical conditions driven by mutations in *GNAQ*, *GNA11*, and *GNA14* genes.

GENE	CDS MUTATION	AA MUTATION	MEDICAL CONDITION
*GNAQ*	c.142_143delinsTT	p.Gly48Leu	Hepatic small vessel neoplasm
	c.143G>T	p.Gly48Val	Cherry hemangioma
	c.548G>A	p.Arg183Gln	SCMs Phacomatosis pigmentovascularis SWS
	c.547C>G	p.Arg183Gly	SCMs
	c.548G>T	p.Arg183Leu	SCMs
	c.623A>C	p.Gln208Pro	Iris and ciliary body melanocytoma
	c.626A>T c.625_626delinsTT	p.Gln209Leu	Congenital hemangioma Anastomosing hemangioma Choroidal nevi Iris and ciliary body melanocytoma Blue nevi Aldosterone-producing adenomas Primary CNS melanocytic neoplasms
	c.626A>G	p.Gln209Arg	Cherry hemangioma Circumscribed choroidal hemangioma
	c.627A>C	p.Gln209His	Congenital hemangioma Cherry hemangioma Anastomosing hemangioma Hepatic small vessel neoplasm
	c.626A>C	p.Gln209Pro	Congenital hemangioma Cherry hemangioma Anastomosing hemangioma Choroidal nevi Blue nevi
*GNA11*	c.546_547delinsTT c.547C>T	p.Arg183Cys	SCMs Phacomatosis pigmentovascularis SWS
	c.548G>A	p.Arg183His	SCMs
	c.547C>A	p.Arg183Ser	Phacomatosis pigmentovascularis
	c.626A>T c.626_627delinsTA c.626_627delinsTT	p.Gln209Leu	Congenital hemangioma Anastomosing hemangioma Choroidal nevi Iris and ciliary body melanocytoma Blue nevi Aldosterone-producing adenomas Primary CNS melanocytic neoplasms
	c.626A>C	p.Gln209Pro	Aldosterone-producing adenomas Primary CNS melanocytic neoplasms
	c.627G>C c.627G>T	p.Gln209His	Cherry hemangioma Anastomosing hemangioma Aldosterone-producing adenomas
*GNA14*	c.614A>T	p.Gln205Leu	Cherry hemangioma Anastomosing hemangioma Hepatic small vessel neoplasm

SWS—Sturge–Weber syndrome; CNS—central nervous system; SCMs—skin capillary malformations.

## 4. Potential Therapeutic Opportunities for UM and Other GNAQ/11-Related Disorders

Cells with mutated GNAQ and GNA11 genes present deregulation in several key signaling pathways, including MAPK (ERK1/2), PI3K/AKT, and YAP/TAZ [29,114,115], hence, potential therapeutic strategies involve targeting these pathways [1].

- **MAPK Pathway:** MEK inhibitors such as selumetinib and trametinib, have been tested to block the ERK1/2 signaling cascade. Although results from clinical trials show limited success, combining MEK inhibitors with other agents like immune checkpoint inhibitors may offer better efficacy [29].
- **YAP/TAZ Pathway:** Since GNAQ/GNA11 mutations activate the Hippo pathway through YAP, drugs targeting YAP or its upstream regulators could be beneficial. Verteporfin, which disrupts YAP-TEAD interactions, is an emerging therapy [29].
- **PKC Inhibition:** Protein kinase C (PKC), downstream of GNAQ/GNA11, is a target of interest, with drugs like AEB071 (sotrastaurin) and LXS196 (darovasertib) under investigation in clinical trials [116,117].

These approaches could reshape the treatment paradigm of UM, shifting focus from conventional UV-based therapies to personalized treatments targeting specific oncogenic drivers. Preclinical studies in vitro and in vivo, as well as early-phase clinical trials, have shown promise for these targeted therapies in improving outcomes for patients with metastatic UM [1]. Beyond UM, targeted therapies could also be explored for other benign and malignant tumors reviewed throughout the article. For example, MEK inhibitors may be useful in managing vascular malformations or benign proliferations like hemangiomas associated with GNAQ/GNA11 mutations [118]. Ongoing studies on the efficacy of targeted treatments in these conditions could expand therapeutic options for patients with other GNAQ/GNA11-driven tumors.

## 5. Future Directions: Potential Histogenetic Role of SOX10-Positive Neural Crest Cells with GNAQ/GNA11 Mutations

The neural crest constitutes an embryonic, multipotent cell population characterized by extensive migration, giving rise to a diverse array of derivatives throughout the body. These include melanocytes, peripheral neurons and glia, as well as craniofacial bone, cartilage, and connective tissue [119]. The embryonic development of neural crest cells and the subsequent differentiation of tissues are intricately regulated by specific transcription factors. Among these, SOX10, a member of the SOX gene family, is particularly remarkable. Located on chromosome 22q13, the *SOX10* (SRY-related HMG-box gene 10) gene encodes a transcription factor essential for the differentiation, migration, and maintenance of tissues derived from neural crest cells. It is pivotal in the development of various tissues, including the central and peripheral nervous systems, melanocytes, chondrocytes, and odontoblasts [120].

SOX10 is a nuclear transcription factor involved in the differentiation of neural crest progenitor cells into melanocytes [121]. During the processes of neural crest induction and migration, SOX10 plays a crucial role in the maintenance of the multipotent state of neural crest cells by inhibiting their differentiation [119]. In diagnostic histopathology, SOX10 immunostaining proves to be a valuable diagnostic tool, due to its high sensitivity in identifying melanocytic and peripheral nerve sheath neoplasms in a variety of neoplasms, including cutaneous adnexal neoplasms, salivary gland neoplasms, breast neoplasms, melanocytic lesions, and peripheral nerve sheath tumors [122].

In a study by Xu et al., the activation of SOX10 by TNF-α through the PI3K/AKT signaling pathway facilitates the differentiation of vascular smooth muscle cells (VSMCs) and promotes vascular inflammation. Silencing SOX10 reduces vascular inflammation and prevents neointimal hyperplasia in RGS5 knockout mice. This study demonstrated that SOX10 acts as a regulator of vascular inflammation and represents a potential control point in inflammation-related vascular diseases [123].

Recently, SOX10-positive perivascular cells were taken under consideration in melanoma studies. It is hypothesized that the perivascular SOX10-positive cells, which express a neural crest marker, are evenly distributed around the vascular adventitia and are oriented parallel to the vascular lumina, may represent cells undergoing Schwannian differentiation linked to mechanoreception. SOX10-positive cells undergo differentiation into pericytes and smooth muscle cells across various tissues and organs, such as the carotid artery, brain, eye, thymus, heart, lung, spleen, and kidney [124]. Studies have identified SOX10-positive perivascular cells in both developing and adult mice, which function as vascular stem cells involved in the development and repair of vascular structures [125]. To our knowledge, there is limited characterization of SOX10-positive perivascular cells in human tissues analyzed through routine pathological examination. We hypothesize that SOX10-positive scattered perivascular cells with GNAQ/GNA11 mutations located in the dermis are the possible source of blue nevi. Further research is required to accurately determine the nature of these cells. The benign nature of SOX10-positive perivascular cells is indicated by their small nuclear size, which often approximates or is smaller than that of small lymphocyte nuclei, their smooth spindled nuclear contours, scant cytoplasm, and evenly spaced perivascular arrangement. These characteristics contrast with metastatic melanoma cells, which typically exhibit nuclear enlargement, pleomorphism, variable amounts of cytoplasm, and irregular distribution [126]. Additionally, benign SOX10-positive perivascular cells are challenging to identify on H&E slides, lack significant atypia, and do not express other melanocytic markers such as Melan-A and HMB45. This helps to prevent potential diagnostic errors that could lead to a false-positive diagnosis of metastatic melanoma [105].

## 6. Conclusions

Uveal melanoma (UM), while exhibiting unique morphological features, shares considerable molecular disruptions with other conditions driven by GNAQ or GNA11 mutations. These mutations are pivotal in abnormal signaling pathways, thereby contributing to the pathogenesis of multiple disorders beyond the UM reviewed in this article. The title “GNAQ/GNA11-related benign and malignant entities—a common histoembryologic origin or a tissue-dependent coincidence”, poses an important question regarding the relationship between the various benign and malignant conditions driven by GNAQ and GNA11 mutations. As presented in this review, these mutations affect a variety of cell types across different tissues, such as melanocytes in uveal melanoma, vascular endothelial cells in hemangiomas, and adrenal cells in aldosterone-producing adenomas. Thus, the specific outcomes of GNAQ and GNA11 mutations seem to depend on several factors, including tissue type, developmental stage, and additional genetic or environmental factors. Especially, the presence of co-occurring mutations or epigenetic changes, such as mutations in BAP1, can influence whether a GNAQ or GNA11 mutation leads to a benign lesion or a malignant tumor. Therefore, evidence suggests that manifestations of these mutations are rather tissue-dependent than the result of a common histoembryologic origin; however, the additional modifying factors undoubtedly play a significant role in determining tumor behavior.

## Data Availability

Not applicable as no datasets were created or generated during the study.
